# Composition and distribution of frogs in Crocker Range Park, Sabah, Malaysia, with a description of a new *Kalophrynus* (Anura, Microhylidae) species

**DOI:** 10.3897/BDJ.13.e157470

**Published:** 2025-09-03

**Authors:** Paul Imbun, Tan Fui Lian, Maklarin Lakim, Luiza Majuakim

**Affiliations:** 1 The Board of Trustees of Sabah Parks, Kota Kinabalu, Sabah, Malaysia The Board of Trustees of Sabah Parks Kota Kinabalu, Sabah Malaysia; 2 Field Museum of Natural History (Amphibians and Reptiles Division), Chicago, Illinois, United States of America Field Museum of Natural History (Amphibians and Reptiles Division) Chicago, Illinois United States of America; 3 Universiti Malaysia Sabah, Institute for Tropical Biology & Conservation, Kota Kinabalu, Sabah, Malaysia Universiti Malaysia Sabah, Institute for Tropical Biology & Conservation Kota Kinabalu, Sabah Malaysia

**Keywords:** frogs, *
Kalophrynus
*, distribution, tropical, protected forest, Crocker Range, Borneo

## Abstract

**Background:**

We monitored five localities within Crocker Range Park, one of the protected forests administered by Sabah Parks in the Malaysian part of Borneo. The sites selected were at various elevations: lowland sites at 260 m and 499 m a.s.l.; montane sites at 1,216 m, 1,260 m and 1,477 m a.s.l. Forty species were encountered in this study. New discoveries were also found during the course of our three-year period survey (October 2003–October 2006).

**New information:**

The discoveries include amongst others, the Sabah endemics of *Meristogenys*, two new records for Sabah: *Sarawakiphrys dringi* (Inger, Stuebing and Tan, 1995), and *Pelophryne rhopophilia* Inger and Stuebing, 1996, as well as a new species, *Kalophrynus minutus* sp. nov., described herein. These findings provide evidence of many unknown species still waiting to be discovered. These discoveries also add significantly to our understanding of the distribution of frog species in Borneo as a whole, showing that species formerly thought to be very restricted geographically actually have much greater and more extensive distributions within Borneo.

## Introduction

The Crocker Range Park (headquarters 05°28’10.8”N, 116°03’22.8”E) with a total area of 1,399 km^2^, covers much of the mountain range lying south of the Kinabalu Park. The Park extends from 150 m to 1,963 m a.s.l. and is covered by both lowland and montane forests. The rainfall recorded at the Park Headquarters is moderate, 1,800–2,200 mm annually and varies monthly although usually no more than two months having less than 100 mm of rain. These drier months may appear at any time during the year. The Park is under the administrative control of Sabah Parks, which is responsible for its protection for the purposes of conservation of its rich flora and fauna. The Park is also important as a water catchment area. Research, much of it carried out by staff of Sabah Parks, proceeds continuously.

Interest in the herpetofauna of the Crocker Range Park (CRP) has been stimulated by several circumstances. First, it is one of the largest areas of relatively undisturbed rain forest in Borneo. Secondly, as most of the knowledge of the submontane and montane herpetofauna of Sabah has been based on the fauna of the Kinabalu Park, it has been a matter of concern to determine to what extent the fauna of Mount Kinabalu is unique to that mountain. Previous samplings of frogs in the CRP have been carried out at relatively low elevations (Inger et al. 2000) or at unspecified elevations (Ramlah et al. 2001, Kueh et al. 2004). The aim of our group was to carry out an inventory of the amphibians at specified low and high elevations. As has been shown at other sites in Borneo (Inger and Voris 1993), repeated sampling of frogs reveals the presence of species not encountered during initial sampling. We decided that our sampling of the frogs in the CRP would be carried out over a three-year period, with frequent sampling over the selected sites. We believed that, by working at a number of stations and for a duration of three years, our inventory would uncover species not previously reported from the CRP and provide a more reliable picture of the fauna at those sites. Our expectations were confirmed by the discovery of new records, species not previously known from CRP and species not previously known from Sabah.

## Materials and methods

### Study site

**Figure 1.**
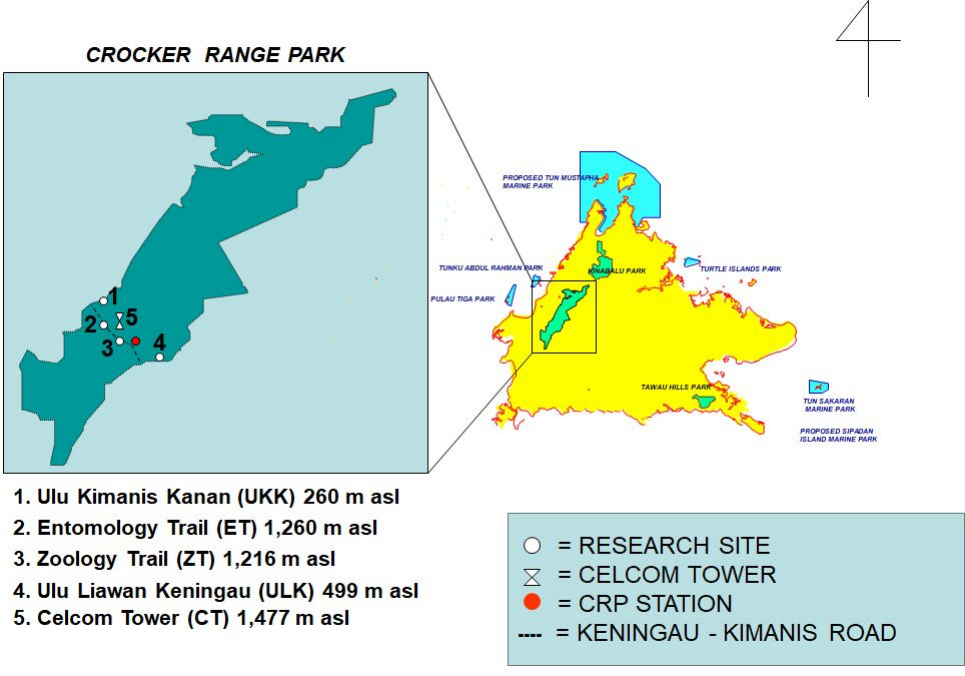
Map showing the sampling locations in Crocker Range Park, Sabah, Malaysian Borneo.

Our fieldwork was conducted at four main sites ranging in elevation from 260 m a.s.l. to approximately 1,500 m a.s.l. (Fig. 1). These sites consisted of:

i) two lowland sites — Ulu Kimanis Kanan (UKK) (05°30’20.7”N, 116°00’22”E; 260 m a.s.l.). At this site we also included tadpole survey stations on Sungai Ulu Kimanis Kanan and along the Ulu Kimanis Trail; and Ulu Liawan Keningau (ULK) (05°24’35”N, 116°07’09”E; 499 m a.s.l.);

ii) two montane sites—Entomology Trail (ET) (05°27’44”N, 116°01’57”E; 1,260 m a.s.l.), situated 14 km from the Park’s administrative office; and Zoology Trail (ZT) (05°27’29”N, 116°04’15”E; 1,216 m a.s.l.).

Two of these sites, UKK and ET, are on the west side of the main ridge of the Crocker Range, whereas two sites, ULK and ZT, are on the east side. In addition to these four sites, we visited one additional site less often — Celcom Tower (CT) (05°28’11”N, 116°03’14”E; 1,477 m a.s.l.).

### Sampling

The samplings of frogs at these sites were carried out periodically (Table 1). At each of the four main sites, we established a forest (non-riparian) transect of 150 m. Along streams of Sungai Ulu Kimanis Kanan (UKK) and Sungai Ulu Liawan Keningau (ULK), we established marked transects of 150 m, with plastic flagging marking at 10 m intervals. On each visit to a site, we worked in each forest and each stream transect for one night, searching for frogs. Transect surveys were sometimes prevented or interrupted by heavy rains.

In addition, we established ten tadpole stations at each stream transect where we searched for frog larvae during the mornings. At the tadpole stations with weak or no current, we used dip nets or short seines with fine mesh. At stations with stronger currents, we used a small portable generator to electro-fish for tadpoles. Tadpoles from each station were placed in separate bags and microhabitat information recorded for the appropriate bag number.

On each visit to a site, we searched for frogs in five forest floor plots (5 m x 5 m) at areas away from streams during daylight hours. The total number of forest floor plots searched during the project was 105. All leaf litter, dead branches and rocks were removed from each forest floor plot as we searched for frogs. All frogs were captured by hand. Each frog caught was placed in a separate numbered bag and microhabitat information recorded with the appropriate bag number.

As we were working in a conservation area, we limited the number of specimens per species preserved. Unless their identifications were in doubt, we released the remainder on site. Specimens were identified immediately at our base camp whenever conditions were possible. Some were photographed before we preserved them. Each specimen was given a tag number before it was euthanised while it was still in the plastic bag. When specimens were dead, we weighed and measured each one. All information was recorded in the field catalogue. The numbered tags were tied on the right upper thigh of each specimen. Specimens were preserved in 10% formalin. Frogs were transferred to 70% ethanol upon arrival at the laboratory at the headquarters of Kinabalu Park. Tadpoles were retained in formalin. All preserved specimens were deposited in the collection of Sabah Parks' zoological museum.

## Taxon treatments

### Kalophrynus minutus

Imbun, Fui Lian, Lakim & Majuakim sp. nov.

8E9AA48C-72C7-5FDA-BC30-6E12D6276CA5

0511980A-A23D-48A5-9BA9-6FCE7CE7D472

#### Materials

- **Type status:** Holotype. **Occurrence:** catalogNumber: SP26180; occurrenceRemarks: collected under dead leaves; recordedBy: Paul Imbun; David Sumpongol; Benedict Butit; Yusili Kumin; Martina Latim; Asmie binti Mian; sex: Male; lifeStage: adult; disposition: in collection; occurrenceID: 0E3A686C-0BEE-55E9-84B9-541CC9D11B5B; **Location:** continent: Borneo; country: Malaysia; stateProvince: Sabah; municipality: Keningau; locality: Zoology Trail, Crocker Range Park; minimumElevationInMeters: 1200; maximumElevationInMeters: 1216; verbatimCoordinates: 5°27’29”N, 116°04’15”E; verbatimLatitude: 5°27’29”N; verbatimLongitude: 116°04’15”E; verbatimCoordinateSystem: degrees minutes seconds; **Identification:** identificationID: SP 26180; **Event:** eventDate: 29 Oct 2006; **Record Level:** institutionID: Sabah Parks; institutionCode: SP; basisOfRecord: PreservedSpecimen
- **Type status:** Paratype. **Occurrence:** catalogNumber: SP20826-27; occurrenceID: 6E4BD4D8-0366-5479-B643-F8EF09C3EF19; **Location:** country: Malaysia; stateProvince: Sabah; municipality: Keningau; locality: Entomology Trail, Crocker Range Park; verbatimElevation: 1216 m; **Event:** eventDate: 5/24/2005; **Record Level:** institutionID: Sabah Parks; institutionCode: SP; basisOfRecord: PreservedSpecimen
- **Type status:** Paratype. **Occurrence:** catalogNumber: SP21204-05; occurrenceID: 3CC9E30F-620A-5C19-9E43-8DCF3A94DFEE; **Location:** country: Malaysia; stateProvince: Sabah; municipality: Keningau; locality: Zoology Trail, Crocker Range Park; verbatimElevation: 1200 m; **Event:** eventDate: 11/9/2005; **Record Level:** institutionID: Sabah Parks; institutionCode: SP; basisOfRecord: PreservedSpecimen
- **Type status:** Paratype. **Occurrence:** catalogNumber: SP21206-08; occurrenceID: 3BCD9032-8D96-5E45-8474-09CB9B5882E7; **Location:** country: Malaysia; stateProvince: Sabah; municipality: Keningau; locality: Entomology Trail, Crocker Range Park; verbatimElevation: 1216 m; **Event:** eventDate: 11/14/2005; **Record Level:** institutionID: Sabah Parks; institutionCode: SP; basisOfRecord: PreservedSpecimen
- **Type status:** Paratype. **Occurrence:** catalogNumber: SP21209-10; occurrenceID: FD777F3F-366C-58FB-9407-5870C86482E5; **Location:** country: Malaysia; stateProvince: Sabah; municipality: Keningau; locality: Zoology Trail, Crocker Range Park; verbatimElevation: 1200 m; **Event:** eventDate: 11/15/2005; **Record Level:** institutionID: Sabah Parks; institutionCode: SP; basisOfRecord: PreservedSpecimen
- **Type status:** Paratype. **Occurrence:** catalogNumber: SP21697-99; occurrenceID: F8FAF680-167F-5BB8-A367-523DDF003A4C; **Location:** country: Malaysia; stateProvince: Sabah; municipality: Keningau; locality: Zoology Trail, Crocker Range Park; verbatimElevation: 1200 m; **Event:** eventDate: 4/8/2006; **Record Level:** institutionID: Sabah Parks; institutionCode: SP; basisOfRecord: PreservedSpecimen
- **Type status:** Paratype. **Occurrence:** catalogNumber: SP21713; occurrenceID: 146D469F-6882-528F-B409-E442FB15FF70; **Location:** country: Malaysia; stateProvince: Sabah; municipality: Keningau; locality: Entomology Trail, Crocker Range Park; verbatimElevation: 1216 m; **Event:** eventDate: 4/10/2006; **Record Level:** institutionID: Sabah Parks; institutionCode: SP; basisOfRecord: PreservedSpecimen
- **Type status:** Paratype. **Occurrence:** catalogNumber: SP26074; occurrenceID: C72A5766-05A9-5DAF-B809-4E005F851A00; **Location:** country: Malaysia; stateProvince: Sabah; municipality: Keningau; locality: Zoology Trail, Crocker Range Park; verbatimElevation: 1200 m; **Event:** eventDate: 9/8/2006; **Record Level:** institutionID: Sabah Parks; institutionCode: SP; basisOfRecord: PreservedSpecimen
- **Type status:** Paratype. **Occurrence:** catalogNumber: SP26076; occurrenceID: 57D2AEBA-E943-501B-B867-39AB08E04D1A; **Location:** country: Malaysia; stateProvince: Sabah; municipality: Keningau; locality: Zoology Trail, Crocker Range Park; verbatimElevation: 1200 m; **Event:** eventDate: 9/8/2006; **Record Level:** institutionID: Sabah Parks; institutionCode: SP; basisOfRecord: PreservedSpecimen
- **Type status:** Paratype. **Occurrence:** catalogNumber: SP26097; occurrenceID: 7E4ABB54-B861-584A-9A8E-B0B77F9A8EA1; **Location:** country: Malaysia; stateProvince: Sabah; municipality: Keningau; locality: Zoology Trail, Crocker Range Park; verbatimElevation: 1200 m; **Event:** eventDate: 9/11/2006; **Record Level:** institutionID: Sabah Parks; institutionCode: SP; basisOfRecord: PreservedSpecimen
- **Type status:** Paratype. **Occurrence:** catalogNumber: SP26179; occurrenceID: FD029B6D-2E0C-5142-8FD0-EC484EB1D40E; **Location:** country: Malaysia; stateProvince: Sabah; municipality: Keningau; locality: Zoology Trail, Crocker Range Park; verbatimElevation: 1200 m; **Event:** eventDate: 10/29/2006; **Record Level:** institutionID: Sabah Parks; institutionCode: SP; basisOfRecord: PreservedSpecimen
- **Type status:** Paratype. **Occurrence:** catalogNumber: SP26181-84; occurrenceID: 6FF9024F-AE20-589B-A3AF-B2958D4FF1AC; **Location:** country: Malaysia; stateProvince: Sabah; municipality: Keningau; locality: Zoology Trail, Crocker Range Park; verbatimElevation: 1200 m; **Event:** eventDate: 10/29/2006; **Record Level:** institutionID: Sabah Parks; institutionCode: SP; basisOfRecord: PreservedSpecimen

#### Description

**Description of holotype**: A small species of *Kalophrynus*, adult males 18.2–20.6 mm (mean ± SE 19.52 ± 0.17, n = 15), adult females 21.1–24.2 mm (mean 22.87, n = 3). Habitus stocky; head not as wide as trunk; head slightly wider than long; snout broadly rounded or obtusely pointed, slightly projecting; nostril lateral, closer to tip of snout than to eye; canthus distinct, weakly rounded, not constricted; lores vertical, not concave; eye diameter slightly shorter than snout length; interorbital about twice width of upper eyelid; tympanum distinct, diameter slightly less than half eye diameter; palate with two crenulate ridges. Fingers and toes with narrow, rounded tips; fleshy web at bases of fingers; extension of fourth finger beyond web shorter than terminal phalanx of third finger; third finger about twice length of second finger; second finger longer than first; fourth finger with one subarticular tubercle, third finger with three; a large, round outer palmar tubercle. Fifth toe shorter than third; foot with fleshy web to tips of first two toes, third and fifth toes with one phalanx free, fourth toe with three phalanges free; toes with low, but large subarticular tubercles; a low, oval inner metatarsal tubercle and an indistinct, round outer one. Skin of dorsum smooth or with few to many low, rounded tubercles; skin of back without deep glandular layer; an oblique dorsolateral row of round, glandular tubercles from above the tympanum to the groin; a U-shaped, thickened skin with dark glandular between the tympanum and the axilla; throat smooth, abdomen weakly granular.

Measurements (mm) and body proportions (holotype in parentheses): SVL 18.2–24.2 mm (19.6), T/SVL 0.427–0.511 (0.485), HW/SVL 0.311–0.415 (0.327).

**Figure 2.**
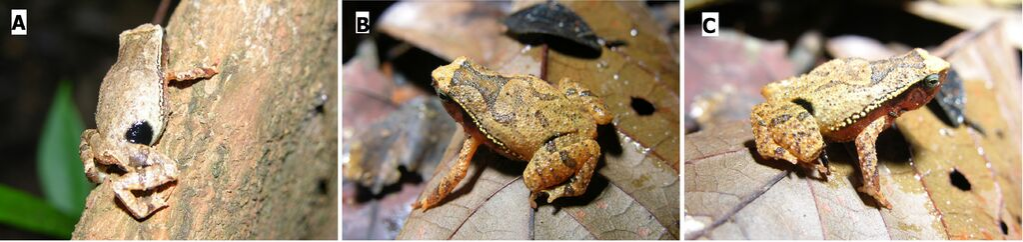
Colouration and dorsolateral pattern of the type specimen (SP26180) of *Kalophrynus minutus* sp. nov. in life. **A** a large, round, black ocellus is visible in the inguinal region; **B–C** show a thick X-shaped pattern over the shoulders and a dorsolateral row of white tubercles with black edging. Photographs: Paul Imbun.

**Colour in life**: Upper body tan to beige depending on lighting of environment, underside of the body lighter colour than the upper body (Fig. 2).

**Figure 3.**
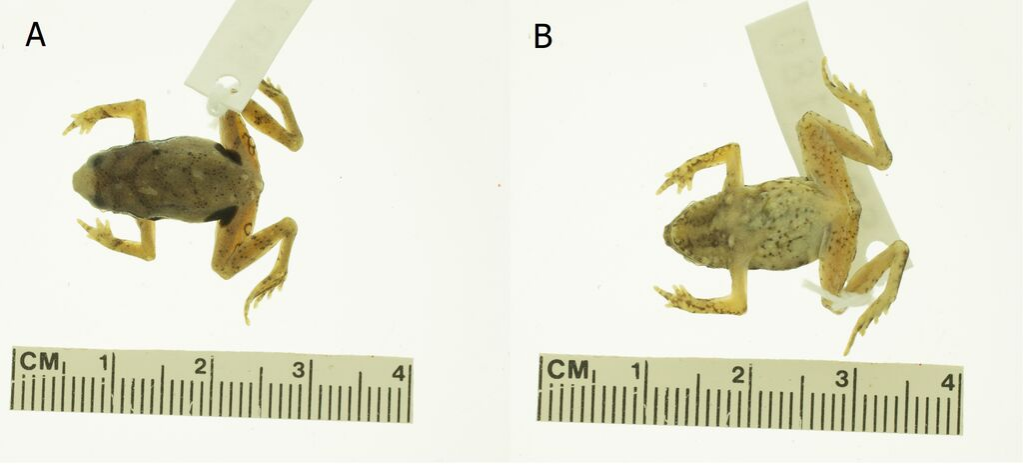
Photographic images of male holotype *Kalophrynus minutus* sp. nov. (SP 26180) in preservation. **A** dorsal view and **B** ventral view. Photographs: Joshua Mata.

**Colour in preservative** (Fig. 3): Above pale tan, uniform or with scattered small black dots or with a thick X-shaped mark over the shoulders with its anterior arms forming a dark interorbital mark and the posterior arms ending just behind shoulders; the dorsolateral row of tubercles white with black edging; ventral surfaces whitish; throat usually with irregular pair of dark longitudinal bands; chest and abdomen with small irregular black markings or small black dots; ventral surfaces of limbs whitish with a few black dots; a large, round, black ocellus in the groin, larger than eye; anterior and posterior faces of thigh with or without one to four black rimmed light spots.

**Secondary sexual characteristics**: Males with slit-like vocal sac openings on each side of the tongue; males without dorsal spinules, nuptial pads or glands on the arm.

#### Diagnosis

A small species of *Kalophrynus*, adults < 25 mm; fourth finger with a single subarticular tubercle, free portion of fourth finger less than length of terminal phalanx of third finger; fifth toe shorter than third; a large black inguinal ocellus.

#### Etymology

The specific epithet is taken from the Latin adjective *minutus* for small, in reference to the new species having the smallest maximum body size in the genus *Kalophrynus*.

#### Distribution

*Kalophrynus minutus* sp. nov., a new species, is only known from two localities on the Crocker Range Park at elevations between 1,200 m a.s.l. to 1,216 m a.s.l. in lower montane tropical forest.

#### Ecology

A terrestrial and sub-fossorial frog species, the adult individuals can be found on dead leaves or the soft soil of the forest floor. Due to its diminutive size and leaf litter colouration, the species can be challenging to detect. Several individuals of the species have been observed in their habitat on each survey. Individuals were found away from ponds or streams or other waterbodies when sampling was done at the type locality. Eggs and tadpoles have never been observed.

#### Taxon discussion

**Comparisons**: The new species was compared with closely-related congeners of *Kalophrynus* in Borneo and Indo-Malesian Regions, based on morphological characters (Suppl. material 1). The comparative morphological data were obtained from literature. Three characters of *Kalophrynus minutus*, new species, distinguish it from most Indo-Malesian species of the genus: the small snout-vent length, the large, solid black ocellus in the groin and the single subarticular tubercle between the palmar tubercle and the tip of the fourth finger. This species resembles *K. eok* (Das and Haas 2003) and *K. subterrestris* (Inger 1966) in having a single subarticular tubercle under the fourth finger, but it differs from those two in having a large, black inguinal ocellus and in size. Dring (1983) said that subarticular tubercles of *K. nubicola* were indistinct or absent, but that species differ from *K. minutus* in lacking an inguinal ocellus. The remaining seven species from the Indo-Malesian region, discussed hereafter, differ from *K. minutus* in having two subarticular tubercles under the fourth finger. In addition, *K. minutus* differs from *K. intermedius* in size [*K. intermedius* females 37–40 mm (Inger 1966)] and in the black ocellus (absent in *K. intermedius*). *Kalophrinus minutus* also differs from *K. baluensis* and *K. heterochirus* in lacking light spots within the inguinal ocellus and in size [females of *K. baluensis* 39 mm (Kiew 1984), females of *K. heterochirus* 30–33 mm (Inger 1966)]. Males of *K. bunguranus*, *K. meizon* and *K. robinsoni* have either spinules on the back or distinct nuptial pads or both, whereas males of *K. minutus* have neither (Inger and Stuebing 2005, Zug 2015). The males of *K. minutus*, on which this statement is based, were caught while calling and hence are considered adult. *Kalophrynus punctatus*, which differs from *K. minutus* in having two subarticular tubercles under the fourth finger, also differs from the new species in having the fifth toe as long as or longer than the third and in lacking a dorsolateral row of black-edged white glandules. *Kalophrynus minutus* is the only one of these Indo-Malesian species in which most individuals have 1–4 black-edged light spots on the rear of the thigh.

### Sarawakiphrys dringi

(Inger, Stuebing & Tan, 1995)

8B042578-B24F-5F58-B324-E0014DA29548

#### Materials

- **Type status:** Other material. **Occurrence:** catalogNumber: SP21832, SP26305; occurrenceRemarks: on forest floor, perching on leaf; individualCount: 2; sex: 1 female, 1 male; lifeStage: adult; disposition: in collection; occurrenceID: C9B22415-35AA-5C1C-B45D-409E1603DF50; **Taxon:** vernacularName: Dring's Horned Frog; **Location:** continent: Borneo; country: Malaysia; stateProvince: Sabah; municipality: Keningau; locality: Celcom Tower, Crocker Range Park; verbatimElevation: 1,477 m; verbatimCoordinates: 05°28’11”N, 116°03’14”E; verbatimCoordinateSystem: degrees minutes seconds; **Identification:** identificationID: SP 21832; SP26305; **Event:** habitat: montane forest

#### Description

Two individuals were collected as specimens. The first is an adult female measuring 43.0 mm snout-vent and holds enlarged, non-pigmented ova. The second is an adult male measuring 51.0 mm and having a black nuptial pad on the first finger. Both lack vomerine teeth, have a single, conical, dermal projection from the upper eyelid, but lack a rostral projection. A single longitudinal dorsolateral fold is present. The female matches all the descriptors in the original description (Inger et al. 1995). The male differs only in that the head is wider than the trunk.

#### Distribution

The distribution of the species is restricted to isolated mountain ranges on Borneo. Presently, it is a first record for CRP and Sabah, with its range previously recorded on Mount Mulu, Sarawak.

#### Ecology

**Figure 4.**
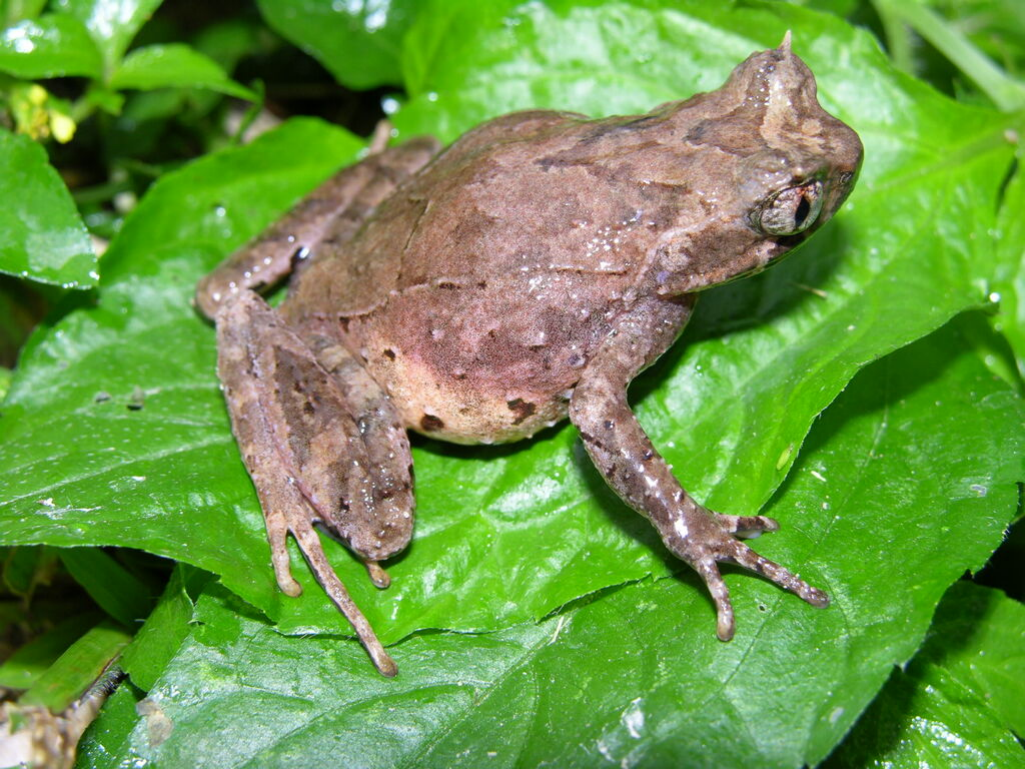
Dorsolateral view of adult female *Sarawakiphrys dringi*, found perching on leaf. Photograph: Paul Imbun.

The species can be found in tropical montane forests at around 1,500 m a.s.l. in CRP and 1,650 m a.s.l. on Mount Mulu. It is a terrestrial species, typically hiding underneath moist leaf litter on the forest floor or perching on leaves (Fig. 4).

### Pelophryne rhopophilia

Inger & Stuebing, 1996

E61C95EB-9344-5A46-9BFC-EC795E5881EB

#### Materials

- **Type status:** Other material. **Occurrence:** catalogNumber: SP 26096; occurrenceRemarks: observed perching on leaf; individualCount: 1; sex: male; lifeStage: adult; disposition: in collection; occurrenceID: 57FFC647-9075-50AF-8A94-CCFA706F12C6; **Taxon:** vernacularName: Lowland Dwarf Toad, Climbing Dwarf Toad; **Location:** continent: Borneo; country: Malaysia; stateProvince: Sabah; municipality: Keningau; locality: Entomology Trail, 14 km from the Crocker Range Park's administrative office; verbatimElevation: 1,216 m; verbatimCoordinates: 05°27’44”N, 116°01’57”E; verbatimCoordinateSystem: degrees minutes seconds; **Event:** eventDate: 9 Sept 2006; habitat: montane forest; **Record Level:** institutionCode: SP; basisOfRecord: PreservedSpecimen

#### Description

An adult male SVL 23.6 mm, with vocal sac opening on right side of floor of mouth and the gular skin stretched and wrinkled. It has a weak nuptial pad of colourless spinules densely packed on the dorsal surface of the first finger, but no mandibular spinules. The outer fingers are expanded and truncate at the tips; web reaches the tip of first finger, one phalanx of second finger is free medially and the outer fingers have two phalanges free. Fleshy web reaches the tips of first three toes laterally, the fifth toe has two phalanges free medially and the fourth toe with three phalanges free; tympanum less than half eye diameter; abdomen black with many small light dots. Three Bornean species of *Pelophryne* have tips of fingers expanded: *P. guentheri* (Boulenger, 1882), *P. signata* (Boulenger, 1895) and *P. rhopophilia* Inger & Stuebing, 1996. The present specimen agrees only with the last species in size [*P. rhopophilia* males 21.7–23.6 mm (Inger and Stuebing 1996)] and absence of mandibular spinules in males.

#### Distribution

As the type (and only recorded) locality is in southern Sarawak, the present record represents a significant extension of the geographic range.

#### Ecology

**Figure 5.**
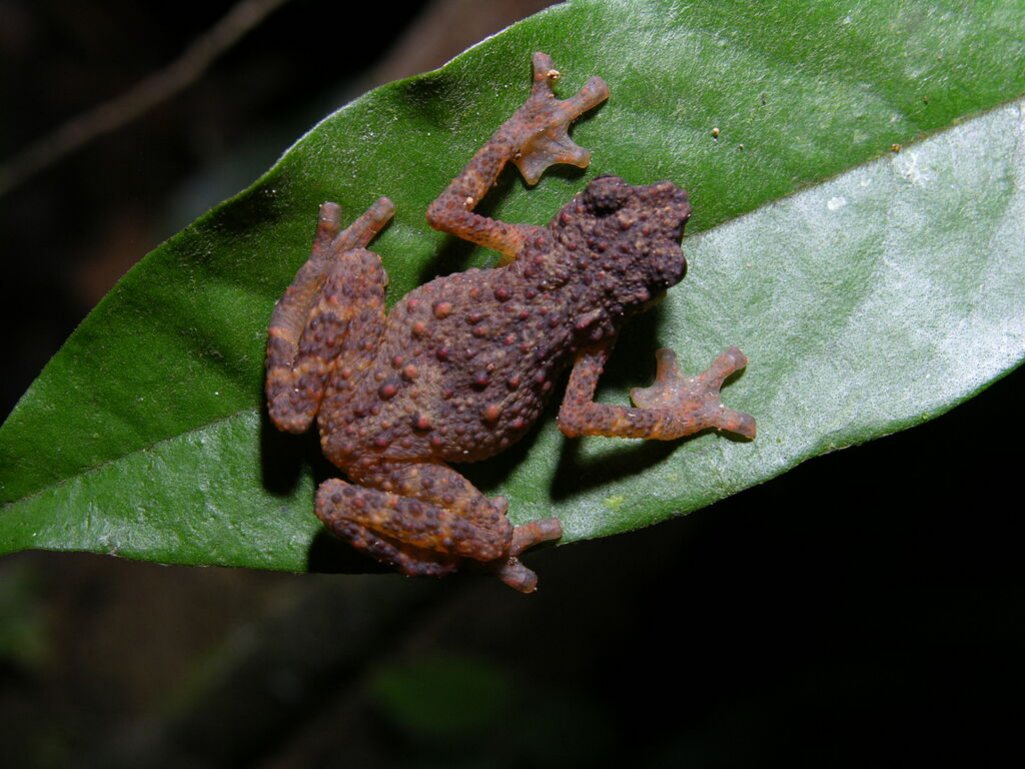
*Pelophryne rhopophilia*, an adult male, perching on leaf. Photograph: Paul Imbun.

Little is known about the natural history of this species other than that it is a montane species. The specimen was found perching on a leaf about 0.5–1.0 m above the ground, in montane forest trail at an elevation of 1,216 m a.s.l. (Fig. 5).

## Checklists

### Species checklist of frogs observed at five localities in the Crocker Range Park, Sabah, Borneo

#### Ansonia hanitschi

Inger, 1960

09DD583C-0FA3-57B3-885C-C0CA6F5CDCED

##### Materials

- **Type status:** Other material. **Occurrence:** occurrenceID: FC4A6429-88EF-5B4B-A01B-5B0D3BEC3750; **Taxon:** kingdom: Animalia; phylum: Chordata; order: Anura; family: Bufonidae; genus: Ansonia; specificEpithet: hanitschi; **Location:** country: Malaysia; stateProvince: Sabah; municipality: Keningau; locality: Crocker Range Park; verbatimElevation: 1477 m; verbatimCoordinates: 05 28 11N, 116 03 14E; verbatimCoordinateSystem: degrees minutes seconds; **Record Level:** institutionCode: SP

#### Ansonia longidigita

Inger, 1960

AB604CD2-D846-5BD0-B07C-05105034EF29

##### Materials

- **Type status:** Other material. **Occurrence:** occurrenceID: 0800BBDE-4BEB-51FA-89A3-4E153716B1EF; **Taxon:** family: Bufonidae; genus: Ansonia; specificEpithet: longidigita; **Location:** country: Malaysia; stateProvince: Sabah; municipality: Keningau; locality: Crocker Range Park; verbatimElevation: 1260-1477 m; minimumElevationInMeters: 1260; maximumElevationInMeters: 1477; verbatimCoordinates: 05 27 44N, 116 01 57E; verbatimCoordinateSystem: degrees minutes seconds; **Record Level:** institutionCode: SP

#### Ansonia platysoma

Inger, 1960

32F4C862-6C95-5C98-B329-52F6966A8070

##### Materials

- **Type status:** Other material. **Occurrence:** occurrenceID: 507D10E0-CF26-5C6C-A00C-03E0E9579A6B; **Taxon:** family: Bufonidae; genus: Ansonia; specificEpithet: platysoma; **Location:** country: Malaysia; stateProvince: Sabah; municipality: Keningau; locality: Crocker Range Park; verbatimElevation: 260-1477 m; minimumElevationInMeters: 260; maximumElevationInMeters: 1477; verbatimCoordinates: 05 24 35N, 116 07 09E; verbatimCoordinateSystem: degrees minutes seconds; **Record Level:** institutionCode: SP

#### Ingerophrynus divergens

(Peters, 1871)

1EB7380B-8F3B-5517-A932-391027EA05B1

##### Materials

- **Type status:** Other material. **Occurrence:** occurrenceID: E63BA616-6B63-5E8D-B242-A1EBE84841B4; **Taxon:** family: Bufonidae; genus: Ingerophrynus; specificEpithet: divergens; **Location:** country: Malaysia; stateProvince: Sabah; municipality: Keningau; locality: Crocker Range Park; verbatimElevation: 260-499 m; minimumElevationInMeters: 260; maximumElevationInMeters: 499; verbatimCoordinates: 05 24 35N, 116 07 09E; verbatimCoordinateSystem: degrees minutes seconds; **Record Level:** institutionCode: SP

#### Leptophryne borbonica

(Tschudi, 1838)

F41024E1-CB68-5992-9E63-9F0E8D2C3A14

##### Materials

- **Type status:** Other material. **Occurrence:** occurrenceID: 06F2E880-4399-56D0-9DC7-FDC34DBDD3AB; **Taxon:** family: Bufonidae; genus: Leptophryne; specificEpithet: borbonica; **Location:** country: Malaysia; stateProvince: Sabah; municipality: Keningau; locality: Crocker Range Park; verbatimElevation: 260-1477 m; minimumElevationInMeters: 260; maximumElevationInMeters: 1477; verbatimCoordinates: 05 27 44N, 116 01 57E; verbatimCoordinateSystem: degrees minutes seconds; **Record Level:** institutionCode: SP

#### Pelophryne rhopophilia

Inger and Stuebing, 1996

AC1F4981-0C65-528A-B032-CB323FA86A93

#### Phrynoidis juxtasper

(Inger, 1964)

AF9CBEEF-0D6F-55ED-B83A-A31C06979E98

##### Materials

- **Type status:** Other material. **Occurrence:** occurrenceID: 5F2D7A5D-24D8-5BF5-9EA8-A0A92BF885EC; **Taxon:** family: Bufonidae; genus: Phrynoidis; specificEpithet: juxtasper; **Location:** country: Malaysia; stateProvince: Sabah; municipality: Keningau; locality: Crocker Range Park; verbatimElevation: 260-499 m; minimumElevationInMeters: 260; maximumElevationInMeters: 499; verbatimCoordinates: 05 24 35N, 116 07 09E; verbatimCoordinateSystem: degrees minutes seconds; **Record Level:** institutionCode: SP

#### Alcalus baluensis

(Boulenger, 1896)

036B3802-3E92-5878-B70D-98CF645B022C

##### Materials

- **Type status:** Other material. **Occurrence:** occurrenceID: 56BC171C-BD00-5A3B-B5AA-110ECE952B01; **Taxon:** family: Ceratobatrachidae; genus: Alcalus; specificEpithet: baluensis; **Location:** country: Malaysia; stateProvince: Sabah; municipality: Keningau; locality: Crocker Range Park; verbatimElevation: 260 m; verbatimCoordinates: 05 30 20.7N, 116 00 22E; verbatimCoordinateSystem: degrees minutes seconds; **Record Level:** institutionCode: SP

#### Limnonectes kuhlii

(Tschudi, 1838)

68A9AD0F-B533-5F7B-AA68-9D0E8D750A30

##### Materials

- **Type status:** Other material. **Occurrence:** occurrenceID: A99A8B94-F4F3-5409-9838-17DA1E880112; **Taxon:** family: Dicroglossidae; genus: Limnonectes; specificEpithet: kuhlii; **Location:** country: Malaysia; stateProvince: Sabah; municipality: Keningau; locality: Crocker Range Park; verbatimElevation: 260-1477 m; minimumElevationInMeters: 260; maximumElevationInMeters: 1477; verbatimCoordinates: 05 27 44N, 116 01 57E; verbatimCoordinateSystem: degrees minutes seconds; **Record Level:** institutionCode: SP

#### Limnonectes leporinus

(Andersson, 1923)

09AB2C63-377D-576C-B4A7-27464D8A5F20

##### Materials

- **Type status:** Other material. **Occurrence:** occurrenceID: A1A048E4-882E-501B-8207-93EC496560FB; **Taxon:** family: Dicroglossidae; genus: Limnonectes; specificEpithet: leporinus; **Location:** country: Malaysia; stateProvince: Sabah; municipality: Keningau; locality: Crocker Range Park; verbatimElevation: 260 m; verbatimCoordinates: 05 30 20.7N, 116 00 22E; verbatimCoordinateSystem: degrees minutes seconds; **Record Level:** institutionCode: SP

#### Limnonectes palavanensis

(Boulenger, 1894)

140C06A2-94E3-5BAE-8D4A-43193EE39A95

##### Materials

- **Type status:** Other material. **Occurrence:** occurrenceID: B6183E94-054D-57CA-8746-BC978C5A802B; **Taxon:** family: Dicroglossidae; genus: Limnonectes; specificEpithet: palavanensis; **Location:** country: Malaysia; stateProvince: Sabah; municipality: Keningau; locality: Crocker Range Park; verbatimElevation: 260-1477 m; minimumElevationInMeters: 260; maximumElevationInMeters: 1477; verbatimCoordinates: 05 27 29N, 116 04 15E; verbatimCoordinateSystem: degrees minutes seconds; **Record Level:** institutionCode: SP

#### Leptobrachella baluensis

Smith, 1931

4E3CA771-522C-56F0-B3C6-16C9FFFC19A7

##### Materials

- **Type status:** Other material. **Occurrence:** occurrenceID: BEE26D6E-6E34-5240-852D-88FCC7B83881; **Taxon:** family: Megophryidae; genus: Leptobrachella; specificEpithet: baluensis; **Location:** country: Malaysia; stateProvince: Sabah; municipality: Keningau; locality: Crocker Range Park; verbatimElevation: 1216-1477 m; minimumElevationInMeters: 1216; maximumElevationInMeters: 1477; verbatimCoordinates: 05 27 29N, 116 04 15E; verbatimCoordinateSystem: degrees minutes seconds; **Record Level:** institutionCode: SP

#### Leptobrachium abbotti

(Cochran, 1926)

42578C23-5661-559D-ACF6-8814AC84D56B

##### Materials

- **Type status:** Other material. **Occurrence:** occurrenceID: C385B1FA-BAEE-5CEF-8F00-461A18DE7B0D; **Taxon:** family: Megophryidae; genus: Leptobrachium; specificEpithet: abbotti; **Location:** country: Malaysia; stateProvince: Sabah; municipality: Keningau; locality: Crocker Range Park; verbatimElevation: 260-499 m; minimumElevationInMeters: 260; maximumElevationInMeters: 499; verbatimCoordinates: 05 30 20.7N, 116 00 22E; verbatimCoordinateSystem: degrees minutes seconds; **Record Level:** institutionCode: SP

#### Leptobrachium montanum

Fischer, 1885

12FB565B-3DA7-51C0-823E-E7186B42B176

##### Materials

- **Type status:** Other material. **Occurrence:** occurrenceID: 4D3974C2-15CF-5F71-B306-8D9C277472C4; **Taxon:** family: Megophryidae; genus: Leptobrachium; specificEpithet: montanum; **Location:** country: Malaysia; stateProvince: Sabah; municipality: Keningau; locality: Crocker Range Park; verbatimElevation: 499-1477 m; minimumElevationInMeters: 499; maximumElevationInMeters: 1477; verbatimCoordinates: 05 24 35N, 116 07 09E; verbatimCoordinateSystem: degrees minutes seconds; **Record Level:** institutionCode: SP

#### Leptolalax dringi

(Dubois, 1987)

A052D0AE-BE08-5D80-99BB-97A24188F4A1

##### Materials

- **Type status:** Other material. **Occurrence:** occurrenceID: 101AFF25-3382-5556-9759-33DB8E48AB83; **Taxon:** family: Megophryidae; genus: Leptolalax; specificEpithet: dringi; **Location:** country: Malaysia; stateProvince: Sabah; municipality: Keningau; locality: Crocker Range Park; verbatimElevation: 260-1477 m; minimumElevationInMeters: 260; maximumElevationInMeters: 1477; verbatimCoordinates: 05 30 20.7N, 116 00 22E; verbatimCoordinateSystem: degrees minutes seconds; **Record Level:** institutionCode: SP

#### Pelobatrachus nasutus

(Schlegel, 1858)

E950B2D6-70BF-55D4-9320-670A326F38D8

##### Materials

- **Type status:** Other material. **Occurrence:** occurrenceID: 43016ABB-9BDA-5FAF-BE7C-0908709D5F8B; **Taxon:** family: Megophryidae; genus: Pelobatrachus; specificEpithet: nasutus; **Location:** country: Malaysia; stateProvince: Sabah; municipality: Keningau; locality: Crocker Range Park; verbatimElevation: 260-1477 m; minimumElevationInMeters: 260; maximumElevationInMeters: 1477; verbatimCoordinates: 05 27 44N, 116 01 57E; verbatimCoordinateSystem: degrees minutes seconds; **Record Level:** institutionCode: SP

#### Sarawakiphrys dringi

(Inger, Stuebing, and Tan, 1995)

78366B1C-8F53-54BD-8033-084638C7F128

#### Chaperina fusca

Mocquard, 1892

864916F2-BF9E-5D88-951B-1E4BE8048184

##### Materials

- **Type status:** Other material. **Occurrence:** occurrenceID: 65EA745B-D779-5A22-93F6-8D9ED7DDE935; **Taxon:** family: Microhylidae; genus: Chaperina; specificEpithet: fusca; **Location:** country: Malaysia; stateProvince: Sabah; municipality: Keningau; locality: Crocker Range Park; verbatimElevation: 1216-1477 m; minimumElevationInMeters: 1216; maximumElevationInMeters: 1477; verbatimCoordinates: 05 27 44N, 116 01 57E; verbatimCoordinateSystem: degrees minutes seconds; **Record Level:** institutionCode: SP

#### Kalophrynus minutus

Imbun, Tan, Lakim, and Majuakim, 2025

3C9CC99F-2DA8-58D1-901A-F1684E05F05A

#### Kaloula pulchra

Gray, 1831

1499E019-19CD-54A3-A989-FC5032E21A91

##### Materials

- **Type status:** Other material. **Occurrence:** occurrenceID: 6964A719-DD95-51CA-94C2-B87754A5382A; **Taxon:** family: Microhylidae; genus: Kaloula; specificEpithet: pulchra; **Location:** country: Malaysia; stateProvince: Sabah; municipality: Keningau; locality: Crocker Range Park; verbatimElevation: 499 m; verbatimCoordinates: 05 24 35N, 116 07 09E; verbatimCoordinateSystem: degrees minutes seconds; **Record Level:** institutionCode: SP

#### Metaphrynella sundana

(Peters, 1867)

E8CEF7CD-F515-5AF6-9C85-412360D8461A

##### Materials

- **Type status:** Other material. **Occurrence:** occurrenceID: F22C98C0-E69D-58F9-BB08-DAED8247098B; **Taxon:** family: Microhylidae; genus: Metaphrynella; specificEpithet: sundana; **Location:** country: Malaysia; stateProvince: Sabah; municipality: Keningau; locality: Crocker Range Park; verbatimElevation: 1260 m; verbatimCoordinates: 05 27 44N, 116 01 57E; verbatimCoordinateSystem: degrees minutes seconds; **Record Level:** institutionCode: SP

#### Huia cavitympanum

(Boulenger, 1893)

B3AC5705-0C37-561F-BE74-AA665B74295D

##### Materials

- **Type status:** Other material. **Occurrence:** occurrenceID: 55E09467-0905-52A8-8D92-E4915166A1DC; **Taxon:** family: Ranidae; genus: Huia; specificEpithet: cavitympanum; **Location:** country: Malaysia; stateProvince: Sabah; municipality: Keningau; locality: Crocker Range Park; verbatimElevation: 260-1216 m; minimumElevationInMeters: 260; maximumElevationInMeters: 1216; verbatimCoordinates: 05 30 20.7N, 116 00 22E; verbatimCoordinateSystem: degrees minutes seconds; **Record Level:** institutionCode: SP

#### Hylarana megalonesa

(Inger, Stuart, and Iskandar, 2009)

A655DC8A-81BD-592B-9703-3461D73767DF

##### Materials

- **Type status:** Other material. **Occurrence:** occurrenceID: 28F7A8D8-A718-5B99-A7CD-9DC76EB4CF69; **Taxon:** family: Ranidae; genus: Hylarana; specificEpithet: megalonesa; **Location:** country: Malaysia; stateProvince: Sabah; municipality: Keningau; locality: Crocker Range Park; verbatimElevation: 499 m; verbatimCoordinates: 05 24 35N, 116 07 09E; verbatimCoordinateSystem: degrees minutes seconds; **Record Level:** institutionCode: SP

#### Meristogenys maryatiae

Matsui, Shimada, and Sudin, 2010

F6109A14-95B0-53A4-BF90-CA155DF0C265

##### Materials

- **Type status:** Other material. **Occurrence:** occurrenceID: 767A80E3-7650-542C-8A49-6135DFBAAEBC; **Taxon:** family: Ranidae; genus: Meristogenys; specificEpithet: maryatiae; **Location:** country: Malaysia; stateProvince: Sabah; municipality: Keningau; locality: Crocker Range Park; verbatimElevation: 260-499 m; minimumElevationInMeters: 260; maximumElevationInMeters: 499; verbatimCoordinates: 05 30 20.7N, 116 00 22E; verbatimCoordinateSystem: degrees minutes seconds; **Record Level:** institutionCode: SP

#### Meristogenys orphnocnemis

(Matsui, 1986)

3D3F06D3-8299-5320-90E7-3ADB8D016AFF

##### Materials

- **Type status:** Other material. **Occurrence:** occurrenceID: 3324110E-4E43-5658-AC76-2591C20C7DD7; **Taxon:** family: Ranidae; genus: Meristogenys; specificEpithet: orphnocnemis; **Location:** country: Malaysia; stateProvince: Sabah; municipality: Keningau; locality: Crocker Range Park; verbatimElevation: 260-1260 m; minimumElevationInMeters: 260; maximumElevationInMeters: 1260; verbatimCoordinates: 05 30 20.7N, 116 00 22E; verbatimCoordinateSystem: degrees minutes seconds; **Record Level:** institutionCode: SP

#### Meristogenys stenocephalus

Shimada, Matsui, Yambun, and Sudin, 2011

8736A3BF-804E-5AD7-B9F7-54CAEBFBE4BD

##### Materials

- **Type status:** Other material. **Occurrence:** occurrenceID: 8DC206A3-B871-5286-9705-9D786BD512EF; **Taxon:** family: Ranidae; genus: Meristogenys; specificEpithet: stenocephalus; **Location:** country: Malaysia; stateProvince: Sabah; municipality: Keningau; locality: Crocker Range Park; verbatimElevation: 499 m; verbatimCoordinates: 05 24 35N, 116 07 09E; verbatimCoordinateSystem: degrees minutes seconds; **Record Level:** institutionCode: SP

#### Meristogenys stigmachilus

Shimada, Matsui, Yambun, and Sudin, 2011

CAB2FC86-F061-513D-A33D-D32E3442AE1C

##### Materials

- **Type status:** Other material. **Occurrence:** occurrenceID: F3491100-D1D9-58B9-AA41-9A712206604E; **Taxon:** family: Ranidae; genus: Meristogenys; specificEpithet: stigmachilus; **Location:** country: Malaysia; stateProvince: Sabah; municipality: Keningau; locality: Crocker Range Park; verbatimElevation: 499 m; verbatimCoordinates: 05 24 35N, 116 07 09E; verbatimCoordinateSystem: degrees minutes seconds; **Record Level:** institutionCode: SP

#### Meristogenys whiteheadi

(Boulenger, 1887)

5D13EBFD-1646-5945-B879-B2B726D5F74E

##### Materials

- **Type status:** Other material. **Occurrence:** occurrenceID: 3D9B1118-732D-58E4-B781-75956115E0D8; **Taxon:** family: Ranidae; genus: Meristogenys; specificEpithet: whiteheadi; **Location:** country: Malaysia; stateProvince: Sabah; municipality: Keningau; locality: Crocker Range Park; verbatimElevation: 499-1260 m; minimumElevationInMeters: 499; maximumElevationInMeters: 1260; verbatimCoordinates: 05 24 35N, 116 07 09E; verbatimCoordinateSystem: degrees minutes seconds; **Record Level:** institutionCode: SP

#### Odorrana hosii

(Boulenger, 1891)

43569054-FA0B-50E0-B694-F37E8CD9AB28

##### Materials

- **Type status:** Other material. **Occurrence:** occurrenceID: EFE03B26-4732-587B-9F48-442E224F70FA; **Taxon:** family: Ranidae; genus: Odorrana; specificEpithet: hosii; **Location:** country: Malaysia; stateProvince: Sabah; municipality: Keningau; locality: Crocker Range Park; verbatimElevation: 260-499 m; minimumElevationInMeters: 260; maximumElevationInMeters: 499; verbatimCoordinates: 05 30 20.7N, 116 00 22E; verbatimCoordinateSystem: degrees minutes seconds; **Record Level:** institutionCode: SP

#### Staurois guttatus

(Günther, 1858)

E233A158-039C-562F-860E-8F8ABC377A4A

##### Materials

- **Type status:** Other material. **Occurrence:** occurrenceID: 95CDA1E5-BB53-516D-93B4-204FE33088AE; **Taxon:** family: Ranidae; genus: Staurois; specificEpithet: guttatus; **Location:** country: Malaysia; stateProvince: Sabah; municipality: Keningau; locality: Crocker Range Park; verbatimElevation: 260-1260 m; minimumElevationInMeters: 260; maximumElevationInMeters: 1260; verbatimCoordinates: 05 30 20.7N, 116 00 22E; verbatimCoordinateSystem: degrees minutes seconds; **Record Level:** institutionCode: SP

#### Staurois latopalmatus

(Boulenger, 1887)

886A9A7C-9746-5DBA-9DB1-1755C68C012D

##### Materials

- **Type status:** Other material. **Occurrence:** occurrenceID: 058A9AA0-C4A2-572E-A617-D7DD08F1BABB; **Taxon:** family: Ranidae; genus: Staurois; specificEpithet: latopalmatus; **Location:** country: Malaysia; stateProvince: Sabah; municipality: Keningau; locality: Crocker Range Park; verbatimElevation: 260-499 m; minimumElevationInMeters: 260; maximumElevationInMeters: 499; verbatimCoordinates: 05 30 20.7N, 116 00 22E; verbatimCoordinateSystem: degrees minutes seconds; **Record Level:** institutionCode: SP

#### Staurois tuberilinguis

Boulenger, 1918

F4D9EF09-6411-599C-A2FC-C535887B0200

##### Materials

- **Type status:** Other material. **Occurrence:** occurrenceID: E4FF1251-2450-5DD9-842A-19DCDAE5E64F; **Taxon:** family: Ranidae; genus: Staurois; specificEpithet: tuberilinguis; **Location:** country: Malaysia; stateProvince: Sabah; municipality: Keningau; locality: Crocker Range Park; verbatimElevation: 1477 m; verbatimCoordinates: 05 28 11N, 116 03 14E; verbatimCoordinateSystem: degrees minutes seconds; **Record Level:** institutionCode: SP

#### Nyctixalus pictus

(Peters, 1871)

9802A66E-C157-5853-8D9D-F89E92F1D64B

##### Materials

- **Type status:** Other material. **Occurrence:** occurrenceID: 4E0D35B3-5E22-5D05-9CEF-B7343AEA5992; **Taxon:** family: Rhacophoridae; genus: Nyctixalus; specificEpithet: pictus; **Location:** country: Malaysia; stateProvince: Sabah; municipality: Keningau; locality: Crocker Range Park; verbatimElevation: 260 m; verbatimCoordinates: 05 30 20.7N, 116 00 22E; verbatimCoordinateSystem: degrees minutes seconds; **Record Level:** institutionCode: SP

#### Philautus aurantium

Inger, 1989

6CAFC807-423C-504E-A563-5AD9B3B9735C

##### Materials

- **Type status:** Other material. **Occurrence:** occurrenceID: AE1760BC-5FC9-5A13-9E49-C545A4B4533D; **Taxon:** family: Rhacophoridae; genus: Philautus; specificEpithet: aurantium; **Location:** country: Malaysia; stateProvince: Sabah; municipality: Keningau; locality: Crocker Range Park; verbatimElevation: 1216-1477 m; minimumElevationInMeters: 1216; maximumElevationInMeters: 1477; verbatimCoordinates: 05 27 44N, 116 01 57E; verbatimCoordinateSystem: degrees minutes seconds; **Record Level:** institutionCode: SP

#### Philautus bunitus

Inger, Stuebing, and Tan, 1995

4EC19798-3AD0-5573-9D43-442D0FE12A9F

##### Materials

- **Type status:** Other material. **Occurrence:** occurrenceID: 2CA3D54C-879C-5C8A-AEF0-7DFF32236163; **Taxon:** family: Rhacophoridae; genus: Philautus; specificEpithet: bunitus; **Location:** country: Malaysia; stateProvince: Sabah; municipality: Keningau; locality: Crocker Range Park; verbatimElevation: 1216-1260 m; minimumElevationInMeters: 1216; maximumElevationInMeters: 1260; verbatimCoordinates: 05 27 44N, 116 01 57E; verbatimCoordinateSystem: degrees minutes seconds; **Record Level:** institutionCode: SP

#### Philautus mjobergi

Smith, 1925

20AF2384-E924-5351-9663-C233D41ADE18

##### Materials

- **Type status:** Other material. **Occurrence:** occurrenceID: C7C35B66-48E4-547E-B528-B489C4753B86; **Taxon:** family: Rhacophoridae; genus: Philautus; specificEpithet: mjobergi; **Location:** country: Malaysia; stateProvince: Sabah; municipality: Keningau; locality: Crocker Range Park; verbatimElevation: 1216-1477 m; minimumElevationInMeters: 1216; maximumElevationInMeters: 1477; verbatimCoordinates: 05 27 29N, 116 04 15E; verbatimCoordinateSystem: degrees minutes seconds; **Record Level:** institutionCode: SP

#### Philautus petersi

(Boulenger, 1900)

285900D2-263E-51EE-B1A6-CF4497955C92

##### Materials

- **Type status:** Other material. **Occurrence:** occurrenceID: E1602E04-5F64-5345-B069-9519BE519AE7; **Taxon:** family: Rhacophoridae; genus: Philautus; specificEpithet: petersi; **Location:** country: Malaysia; stateProvince: Sabah; municipality: Keningau; locality: Crocker Range Park; verbatimElevation: 260-1477 m; minimumElevationInMeters: 260; maximumElevationInMeters: 1477; verbatimCoordinates: 05 27 44N, 116 01 57E; verbatimCoordinateSystem: degrees minutes seconds; **Record Level:** institutionCode: SP

#### Polypedates macrotis

(Boulenger, 1891)

76F91A20-375C-55C8-8493-EF08AF20E50F

##### Materials

- **Type status:** Other material. **Occurrence:** occurrenceID: F8A6FCAA-06E3-54CC-8A4B-E59C2E046ED4; **Taxon:** family: Rhacophoridae; genus: Polypedates; specificEpithet: macrotis; **Location:** country: Malaysia; stateProvince: Sabah; municipality: Keningau; locality: Crocker Range Park; verbatimElevation: 260-499 m; minimumElevationInMeters: 260; maximumElevationInMeters: 499; verbatimCoordinates: 05 30 20.7N, 116 00 22E; verbatimCoordinateSystem: degrees minutes seconds; **Record Level:** institutionCode: SP

#### Philautus macroscelis

(Boulenger, 1896)

20DFF873-F8ED-58C7-9CB7-1B1CBBB5CB12

##### Materials

- **Type status:** Other material. **Occurrence:** occurrenceID: AC794FAE-D9F5-5813-B022-4FBEBA0018CF; **Taxon:** family: Rhacophoridae; genus: Philautus; specificEpithet: macroscelis; **Location:** country: Malaysia; stateProvince: Sabah; municipality: Keningau; locality: Crocker Range Park; verbatimElevation: 1477 m; verbatimCoordinates: 05 28 11N, 116 03 14E; verbatimCoordinateSystem: degrees minutes seconds; **Record Level:** institutionCode: SP

#### Rhacophorus nigropalmatus

Boulenger, 1895

830650DD-FA3B-5239-B398-22E2FBF8463E

##### Materials

- **Type status:** Other material. **Occurrence:** occurrenceID: 3B2C8994-36BB-5049-A8AE-91A14C57A87A; **Taxon:** family: Rhacophoridae; genus: Rhacophorus; specificEpithet: nigropalmatus; **Location:** country: Malaysia; stateProvince: Sabah; municipality: Keningau; locality: Crocker Range Park; verbatimElevation: 1216 m; verbatimCoordinates: 05 27 29N, 116 04 15E; verbatimCoordinateSystem: degrees minutes seconds; **Record Level:** institutionCode: SP

## Analysis

**Species composition and new records**: Forty species of frogs were observed. Table 2 presents a summary of collecting effort and numbers of species and individuals observed at lowland and montane sites. A total of 119 transects were installed in both lowland and montane sites during the three years sampling period resulting in 575 observations. Twenty-seven species were found only at the lowland localities (UKK and ULK), whilst 15 species were encountered only at high (> 1,400 m a.s.l.) elevation locality (CT) (Table 3). Two of these 15 species, *Chaperina fusca* and *Staurois tuberilinguis*, have been found elsewhere at below 500 m a.s.l. in the CRP (Inger et al. 2000).

Eight species constitute new records for the CRP. Of these eight species, *Ingerophrynus divergens*, *Kaloula pulchra*, *Occidozyga laevis* and *Rhacophorus nigropalmatus* have been found at other sites in Sabah. A new, diminutive species of *Kalophrynus*, is also recorded and described herein. We also recovered *Meristogenys* species endemic to Sabah, *M. maryatiae*, *M. stigmachilus* and *M. stenocephalus*. The three frogs collected at ULK belong to the *Meristogenys whiteheadi* group as defined by Shimada et al. (2011). One (SP20403 ♂, SVL 51.5 mm, HW 18.8 mm) has lip pattern 1-b (using the system of Shimada et al. (2011)) corresponding to *M. stigmachilus*. The other two have lip patterns 3 (SP21196 ♀, SVL 77.0 mm, HW 26.2 mm) and 4 (SP20676 ♂, SVL 51.9 mm, HW 16.6 mm), respectively, patterns falling within the range of variation of *M. stenocephalus*.

Of the eight new records for CRP, two species also constitute new records for Sabah; these are *Sarawakiphrys dringi* and *Pelophryne rhopophilia*. Until now, these species were known to occur only at their type localities in Sarawak (Inger and Stuebing 2005, Inger et al. 2017). The discovery of these frogs at CRP has expanded their geographical ranges in Borneo highlighting the importance of CRP as a significant area for biodiversity conservation and the potential for further undiscovered species in the region.

## Discussion

The 40 species observed in this study and the 25 reported by other authors (Inger et al. 2000, Ramlah et al. 2001, Kueh et al. 2004) bring the total known anuran fauna of the CRP to 63. Each new survey in CRP discovers additions to the known fauna, which makes it likely that we have not yet reached the full count of frogs for this Park. The new discoveries increase the conservation importance of this Park. It is not possible to say now how many species of frogs may actually occur in the CRP, but surely there are many more than previously thought. The new discoveries made during our survey include both lowland *(Ingerophrynus divergens*) and montane (*Sarawakiphrys dringi*) species. The recently-described species *Meristogenys maryatiae* and the new species reported herein, *Kalophrynus minutus* sp. nov., are evidence of existing unknowns waiting to be discovered. These discoveries also add significantly to our understanding of the distribution of species in Borneo as a whole, showing that species that were formerly thought to be very restricted geographically, for example, *Sarawakiphrys dringi*, actually have much greater and more extensive distributions within Borneo. Our survey also demonstrates that the lowland portion of the fauna in CRP is likely to be much larger than previously thought. An interesting unanswered question is whether some of the distinctive high elevation species known only from Mount Kinabalu, such as *Pelophryne misera* and *Ansonia fuliginea*, will also be discovered in the CRP. Eight of the montane (occurring above 1,000 m a.s.l.) species known from Kinabalu have not yet been found in the CRP. If future work in CRP does discover these species, the future for these distinctive species will be more hopeful.

**Taxonomic remarks and future work**

Even though the description of the new species, *Kalophrynus minutus* sp. nov., is based solely on morphological data, the distinctive combination of characters observed in *Kalophrynus minutus* sp. nov. delineates it from its congeners and provides a clear justification for its recognition as a new species. We acknowledge that molecular phylogenetic data would further support its taxonomic placement and divergence from congeners. However, due to specimen preservation, logistic and field limitations, DNA sampling was not feasible during the survey. Additional specimens from the type locality or nearby areas may allow future supplementation for the description of *Kalophrynus minutus* sp. nov. with molecular phylogenetic data. This would help clarify its evolutionary relationships within its congeners.

## Supplementary Material

XML Treatment for Kalophrynus minutus

XML Treatment for Sarawakiphrys dringi

XML Treatment for Pelophryne rhopophilia

XML Treatment for Ansonia hanitschi

XML Treatment for Ansonia longidigita

XML Treatment for Ansonia platysoma

XML Treatment for Ingerophrynus divergens

XML Treatment for Leptophryne borbonica

XML Treatment for Pelophryne rhopophilia

XML Treatment for Phrynoidis juxtasper

XML Treatment for Alcalus baluensis

XML Treatment for Limnonectes kuhlii

XML Treatment for Limnonectes leporinus

XML Treatment for Limnonectes palavanensis

XML Treatment for Leptobrachella baluensis

XML Treatment for Leptobrachium abbotti

XML Treatment for Leptobrachium montanum

XML Treatment for Leptolalax dringi

XML Treatment for Pelobatrachus nasutus

XML Treatment for Sarawakiphrys dringi

XML Treatment for Chaperina fusca

XML Treatment for Kalophrynus minutus

XML Treatment for Kaloula pulchra

XML Treatment for Metaphrynella sundana

XML Treatment for Huia cavitympanum

XML Treatment for Hylarana megalonesa

XML Treatment for Meristogenys maryatiae

XML Treatment for Meristogenys orphnocnemis

XML Treatment for Meristogenys stenocephalus

XML Treatment for Meristogenys stigmachilus

XML Treatment for Meristogenys whiteheadi

XML Treatment for Odorrana hosii

XML Treatment for Staurois guttatus

XML Treatment for Staurois latopalmatus

XML Treatment for Staurois tuberilinguis

XML Treatment for Nyctixalus pictus

XML Treatment for Philautus aurantium

XML Treatment for Philautus bunitus

XML Treatment for Philautus mjobergi

XML Treatment for Philautus petersi

XML Treatment for Polypedates macrotis

XML Treatment for Philautus macroscelis

XML Treatment for Rhacophorus nigropalmatus

FC38FF4A-2141-5A51-82B0-0D79DFC77C89 10.3897/BDJ.13.e157470.suppl1 Supplementary material 1 Distinguishing morphological characters of *Kalophrynus* species in Borneo and nearby regions, compared to *Kalophrynus minutus* sp. nov. Data type Morphological Brief description The new species, *Kalophrynus minutus*, was compared with closely-related congeners of *Kalophrynus* in Borneo and Indo-Malesian Regions, based on morphological characters. The comparative morphological data were obtained from literature. File: oo_1317380.docx https://binary.pensoft.net/file/1317380 Paul Imbun, Tan Fui Lian, Maklarin Lakim & Luiza Majuakim

## Figures and Tables

**Table 1.** The sampling sites in Crocker Range Park and periods sampled in 2003–2006 (*Sites abbreviation: UKK = Ulu Kimanis Kanan; ULK = Ulu Liawan Keningau; ZT = Zoology Trail; ET = Entomology Trail; CT = Celcom Tower).

**Sampling period**	Sites*				
	**UKK**	**ULK**	**ZT**	**ET**	**CT**
October 2003	+	+	+	+	
April 2004	+	+	+	+	
August 2004	+	+	+	+	
November 2004	+	+	+	+	
February–March 2005	+	+	+	+	
May 2005	+	+	+	+	
August–September 2005	+	+	+	+	+
November 2005	+	+	+	+	+
April 2006			+	+	+
September 2006			+	+	
October 2006			+	+	

**Table 2.** Summary of collecting effort and numbers of species and individuals of frogs observed.

**Forest habitat**	**Number of transect surveys**	**Number of individuals**	**Number of species**
Lowland (260 and 499 m a.s.l.)	42	211	26
Montane (1,200–1,477 m a.s.l.)	77	364	26

**Table 3.** Species and numbers of individuals observed at five localities in the Crocker Range Park (^1^Abbreviations of localities: UKK—Ulu Kimanis Kanan; ULK—Ulu Liawan Keningau; ZT—Zoology Trail; ET—Entomology Trail; CT—Celcom Tower. *New species described in present paper. ** New record for Sabah).

Taxon	Localities^1^ Elevation (m a.s.l.)				
	UKK	ULK	ZT	ET	CT
	260	499	1,216	1,260	1,477
** BUFONIDAE **					
*Ansonia hanitschi* Inger, 1960					1
*Ansonia longidigita* Inger, 1960	1			8	
*Ansonia platysoma* Inger, 1960	1		1	2	4
*Ingerophrynus divergens* (Peters, 1871)	4	1			
*Leptophryne borbonica* (Tschudi, 1838)	1		11	22	1
*Pelophryne rhopophilia* Inger and Stuebing, 1996**				1	
*Phrynoidis juxtasper* (Inger, 1964)	7	12			
** CERATOBATRACHIDAE **					
*Alcalus baluensis* (Boulenger, 1896)	1				
** DICROGLOSSIDAE **					
*Limnonectes kuhlii* (Tschudi, 1838)	3		8	23	25
*Limnonectes leporinus* (Andersson, 1923)	10				
*Limnonectes palavanensis* (Boulenger, 1894)	2		26	15	3
** MEGOPHRYIDAE **					
*Leptobrachella baluensis* Smith, 1931			1	1	1
*Leptobrachium abbotti* (Cochran, 1926)	4	2			
*Leptobrachium montanum* Fischer, 1885		1	2	2	7
*Leptolalax dringi* (Dubois, 1987)	6	13		4	1
*Pelobatrachus nasutus* (Schlegel, 1858)	1			3	1
*Sarawakiphrys dringi* (Inger, Stuebing, and Tan, 1995)**					5
** MICROHYLIDAE **					
*Chaperina fusca* Mocquard, 1892			47	20	
*Kalophrynus minutus* sp. nov.*			19	3	
*Kaloula pulchra* Gray, 1831		1			
*Metaphrynella sundana* (Peters, 1867)				1	
** RANIDAE **					
*Huia cavitympanum* (Boulenger, 1893)	3		1		
*Hylarana megalonesa* (Inger, Stuart, and Iskandar, 2009)		1			
*Meristogenys maryatiae* Matsui, Shimada, and Sudin, 2010	2	1			
*Meristogenys orphnocnemis* (Matsui, 1986)	18	45	1	4	
*Meristogenys stenocephalus* Shimada, Matsui, Yambun, and Sudin, 2011		2			
*Meristogenys stigmachilus* Shimada, Matsui, Yambun, and Sudin, 2011		1			
*Meristogenys whiteheadi* (Boulenger, 1887)		1		1	
*Odorrana hosii* (Boulenger, 1891)	7	2			
*Staurois guttatus* (Günther, 1858)	1			1	
*Staurois latopalmatus* (Boulenger, 1887)	37	14			
*Staurois tuberilinguis* Boulenger, 1918					3
** RHACOPHORIDAE **					
*Nyctixalus pictus* (Peters, 1871)	1				
*Philautus aurantium* Inger, 1989			1	11	2
*Philautus bunitus* Inger, Stuebing, and Tan, 1995			2	11	
*Philautus mjobergi* Smith, 1925			4	8	6
*Philautus petersi* (Boulenger, 1900)	3		4	22	1
*Polypedates macrotis* (Boulenger, 1891)	1	1			
*Philautus macroscelis* (Boulenger, 1896)					11
*Rhacophorus nigropalmatus* Boulenger, 1895			4		
