# Supplementary material for: Composition and distribution of frogs in Crocker Range Park, Sabah, Malaysia, with a description of a new Kalophrynus (Anura, Microhylidae) species
Source: Biodivers Data J. 2025 Sep 3;13:e157470. doi: 10.3897/BDJ.13.e157470 (PMC12423637; doi:10.3897/BDJ.13.e157470)
Supplement: Supplementary material 1 — Distinguishing morphological characters of Kalophrynus species in Borneo and nearby regions, compared to Kalophrynus minutus sp. nov. [file bdj-13-e157470-s001.docx]

| Species | Type locality | Altitude (m asl) | Snout Vent Length (SVL) (mm) | | Inguinal ocellus | | | Subarticular tubercles under 4th finger | Male sex characters | | Spinules on back | Extent of 3rd and 5th toes | References |
| --- | --- | --- | --- | --- | --- | --- | --- | --- | --- | --- | --- | --- | --- |
|  |  |  | Male | Female | Present | Colour | Light spots within |  | Distinct nuptial pads | Spinules on nuptial pad |  |  |  |
| *Kalophrynus minutus* sp. nov. | Crocker Range Park, Sabah | 1,200–1,216 | 18.2–20.6 | 21.1–24.2 | yes | black with white edges | – | 1 | – | – | – | 3>5 | described in this paper |
| *Kalophrynus baluensis* Kiew, 1984 | Mount Kinabalu, Sabah | 1,400–1800 | 33–36 | 39–47 | yes | yellow with dark edges | + | 2 | n/k | n/k | – | 3>5 | Inger & Stuebing (2005), Zug (2015) |
| *Kalophrynus bunguranus* (Günther, 1895) | Bunguran, or Great Natuna Island, Indonesia | n/k | 22–23 | 25–27 | yes | black | – | 2 | – | – | + | 3>5 | Inger (1966), Zug (2015) |
| *Kalophrynus eok* Das and Haas, 2003 | Bario (Kelabit Highlands), Sarawak | 1,050 | 26.3 | n/k | no | none | – | 1 | + | – | – | 3>5 | Das & Haas (2003) |
| *Kalophrynus heterochirus* Boulenger, 1900 | Borneo | 0–1,200 | 24–27 | 30–33 | yes | bluish-white with black edges | + | 2 | – | – | – | 3>5 | Inger (1966), Inger & Stuebing (2005) |
| *Kalophrynus intermedius* Inger, 1966 | Third Division, Sarawak | below 300 | 27 | 38–41 | no | none | – | 2 | n/k | n/k | – | 3>5 | Inger (1966), Inger & Stuebing (2005) |
| *Kalophrynus meizon* Zug, 2015 | Bintulu Division, Sarawak | 800 and below | 37–50 | 35–57 | yes | black with white edges | – | 2 | + | + | + | 3>5 | Zug (2015) |
| *Kalophrynus nubicola* Dring, 1983 | Gunung Mulu, Sarawak | 1,300–2,200 | 14–24 | 21–24 | no | none | – | indistinct or absent | + | + | + | 3≈5 | Dring (1983) |
| *Kalophrynus punctatus* Peters, 1871 | Borneo | lowland to lower montane zone | 22–28 | n/k | no | none | – | 2 | – | – | – | 3<5 | Inger (1966), Inger & Stuebing (2005), Zug (2015) |
| *Kalophrynus robinsoni* Smith, 1922 | Pahang, West Malaysia | 1,005 | 17–18 | 18 | no | none | – | – | + | + | – | 3>5 | Inger (1966), Zug (2015) |
| *Kalophrynus subterrestris* Inger, 1966 | Bintulu, Sarawak | lowland | 21– 23 | 26–27 | with or without | black | – | 1 | – | – | – | 3>5 | Inger (1966), Inger & Stuebing (2005), Zug (2015) |
